# Spin-wave mode coupling in the presence of the demagnetizing field in cobalt-permalloy magnonic crystals

**DOI:** 10.1038/s41598-024-74923-2

**Published:** 2024-10-03

**Authors:** S. Mamica

**Affiliations:** Faculty of Physics, ISQI, A. Mickiewicz University in Poznań, ul. Uniwersytetu Poznańskiego 2, Poznań, 61-614 Poland

**Keywords:** Magnonic crystals, Spin-waves, Mode coupling, Hybridization, Demagnetizing field, Condensed-matter physics, Nanoscale materials, Magnetic properties and materials, Surfaces, interfaces and thin films

## Abstract

We present the results of studies on the non-uniform frequency shift of spin wave spectrum in a two-dimensional magnonic crystal of cobalt/permalloy under the influence of external magnetic field changes. We investigate the phenomenon of coupling of modes and, as a consequence, their hybridization. By taking advantage of the fact that compressing the crystal structure along the direction of the external magnetic field leads to an enhancement of the demagnetizing field, we analyse its effect on the frequency shift of individual modes depending on their concentration in Co. We show that the consequence of this enhancement is a shift in the coupling of modes towards higher magnetic fields. This provides a potential opportunity to design which pairs of modes and in what range of fields hybridization will occur.

## Introduction

Magnetic materials exhibit many interesting properties, which result, among other things, from the coexistence of various types of interactions. For example, in finite ferromagnetic systems, such as magnetic dots, at least two types of interactions occur, which compete with each other, namely exchange and dipolar interactions. This leads to the formation of peculiar magnetization configurations in the ground state, such as labyrinthine^1,2^or vortex structures^3–5^. Additionally, the presence of Dzyaloshinskii-Moriya interactions (DMI) or specific anisotropy (even without DMI) results in the appearance of structures of skyrmion types^6–11^.

Of course, the coexistence of different types of interactions affects not only static magnetization but also its dynamics. It is a source of richness in the spectrum of spin waves, or magnetization excitations^12–17^. For example, in magnetic nano-dots in a vortex state, a change in the system’s size leads to a change in the order of eigenmodes^18^. Consequently, excitations with similar symmetries approach each other (in frequency domain), which in turn results in their interaction (coupling) and the resulting hybridization^19^. Another example of such phenomena is the non-uniform frequency shift of spin waves under the influence of changes in the external magnetic field observed in various magnetic systems^20–26^. In periodic structures, such as magnonic crystals, this effect can lead to the opening of a complete band gap^27,28^.

Magnonic crystals (MCs), as periodic composites, are the magnetic counterpart of structures such as photonic^29^or phononic crystals^30,31^. They have been an important branch of research for nearly three decades. The origins of research on MCs date back to 1996 when the Plane Wave Method was applied to calculate the spectrum of spin waves in a two-dimensional magnetic composite^32^. The term “magnonic crystal” was first used in 2002^33^. Due to the relatively easy fabrication, one- and two-dimensional MCs are particularly popular^34–41^, but three-dimensional MCs are also being considered^42–45^, and new fabrication methods are creating opportunities for their experimental investigation^46–49^.

The systems studied in this work are two-dimensional (2D) MCs, which are characterized by periodicity in the plane. Typical representatives of 2D MCs are bicomponent MCs and antidot lattices (ADLs)^36^. An ADL is formed by creating a regular array of holes, called antidots, in a thin film of magnetic material. If the antidots are filled with a magnetic material different from that of the thin film, a regular array of magnetic dots from one material embedded in a matrix of another material is obtained. This system is referred to as a bicomponent 2D MC. Spin waves are excited only in the magnetic material, so for ADLs, it occurs solely in the matrix. In contrast, for 2D MCs, they are excited in both materials simultaneously—with different intensities in each. This causes both the spin wave spectrum in bicomponent MCs and their response to an external magnetic field to differ from those in ADLs. In this work, we investigate bicomponent 2D MCs, briefly denoting them as 2D MCs. Another way to achieve 2D periodicity is to overlay a regular array of dots, not necessarily magnetic, on the surface of a homogeneous thin magnetic film. The presence of the additional material locally modifies the magnetic properties of the thin film, resulting in a 2D MC^50^.

Research on MCs has initiated the emergence of a new field known as magnonics, which is the equivalent of traditional electronics, utilizing spin waves as carriers of information. In recent years, there has been a significant surge of interest in this field^51^. This increasing interest is primarily due to the demonstration of several elementary magnonic devices, the development of which aims to enable computations directly on spin waves. An example of such a device is the spin-wave diode. This is a relatively simple system consisting of two or three magnetic strips (1D waveguides) that are positioned close enough together over a certain length such that the propagating spin waves couple through dipole interactions^52^or through interfacial Dzyaloshinskii-Moriya interaction^53^. In both cases, the effect of non-reciprocal hybridization occurs, allowing the coupling of spin waves to only happen in one direction of propagation. Another device using the same mechanism is the circulator, in which the signal exiting from one port is always directed to the nearest port, following the specified rotation direction^53^. Another example of a magnonic device is the delay-coupling loop, which utilizes the resonant scattering of spin waves^54,55^. In this configuration, the efficiency of resonant scattering is determined by the coupling between the propagating waves and the discrete modes of the resonator. One of the applications of such resonator systems could be magnonic artificial neural networks^56^.

In all of these systems, the basis of their operation is the coupling between different types of wave-spin modes and the accompanying hybridization. Thus the phenomenon of hybridization itself is particularly important from an application standpoint, i.e., due to the ability to manipulate the propagation of spin waves using physical quantities such as magnetic field, electric field, or temperature^57–66^. Hence, there has been a significant increase in interest in studying the coupling between spin waves and other types of excitations, as well as between different modes of spin waves. Particularly promising are methods that allow for intervention in the properties of hybridizing modes in operando^60,61,65,66^, and those that allow for the reduction of the damping of spin waves^67^.

In 2D systems, the hybridization of spin waves has been experimentally observed in both bi-component MCs and ADLs. For example, in the work^68^, both permalloy (Py) ADLs with alternating hole diameters and 2D MCs consisting of Co disks partially embedded in a Py ADL were studied. In both systems, hybridization between different modes of spin waves was observed. It was noted, for instance, that for ADLs, the presence of holes with alternating diameters induces a high degree of hybridization in the profile of the localized mode. In contrast, for the 2D MC mode, which is localized for short wave vectors, as the wave vector increases, it does not remain localized but spreads throughout the entire matrix due to hybridization. An interesting result was obtained in the work^69^, where ADLs based on a Co/Pd multilayer were investigated. Due to the fabrication process, each antidot is surrounded by a rim with magnetic parameters changing gradually. Two types of modes were observed: bulk and rim, among others. And for some particular ranges of the external magnetic field, bulk-rim hybridization was observed. Even without such a rim, at the edges of holes in ADLs, a strong demagnetizing field appears, and this demagnetizing region can be a source of mode hybridization^70^.

In this work, we present the results of our studies on the phenomenon of non-uniform frequency shift of spin wave spectra in two-dimensional magnonic crystals (2D MCs). In particular, we are interested in the influence of demagnetizing field on the interaction of spin waves and the related hybridization. Changes in the demagnetization field are achieved by squeezing the MC in the plane in the external magnetic field direction. In our previous work^71^, we investigated changes in the energy concentration of spin-wave excitations under such squeezing and the resulting enhancement of the demagnetization field, as well as their consequences for the existence of a magnonic band gap. We also noticed the hybridization of modes within a small range of analysed structure ratio values. In this work, we study the influence of the demagnetization field enhancement on the hybridization phenomenon. We show that as a result of this enhancement, the coupling of modes shifts towards higher magnetic fields, potentially allowing for the design of which mode pairs and within what range of fields will undergo hybridization.

We consider a structure possible to be fabricated using currently available technology, made of popular ferromagnetic materials (cobalt and permalloy). In our theoretical studies, we use the plane wave method (PWM), successfully applied to calculate spin wave spectra in 2D MCs. We describe the method and the analysed structure in Section “The model and the method”. In Section “Results and discussion”, we analyse the results obtained for a hexagonal 2D MC structure. We are interested in the effects of non-uniform frequency shift with changes in the external magnetic field, and in particular, the appearance of coupling between modes and the resulting hybridization. Next, results for structures exhibiting enhanced demagnetization field are discussed. We conclude the work with a summary in Section “Conclusions”.

## The model and the method

**Fig. 1:**
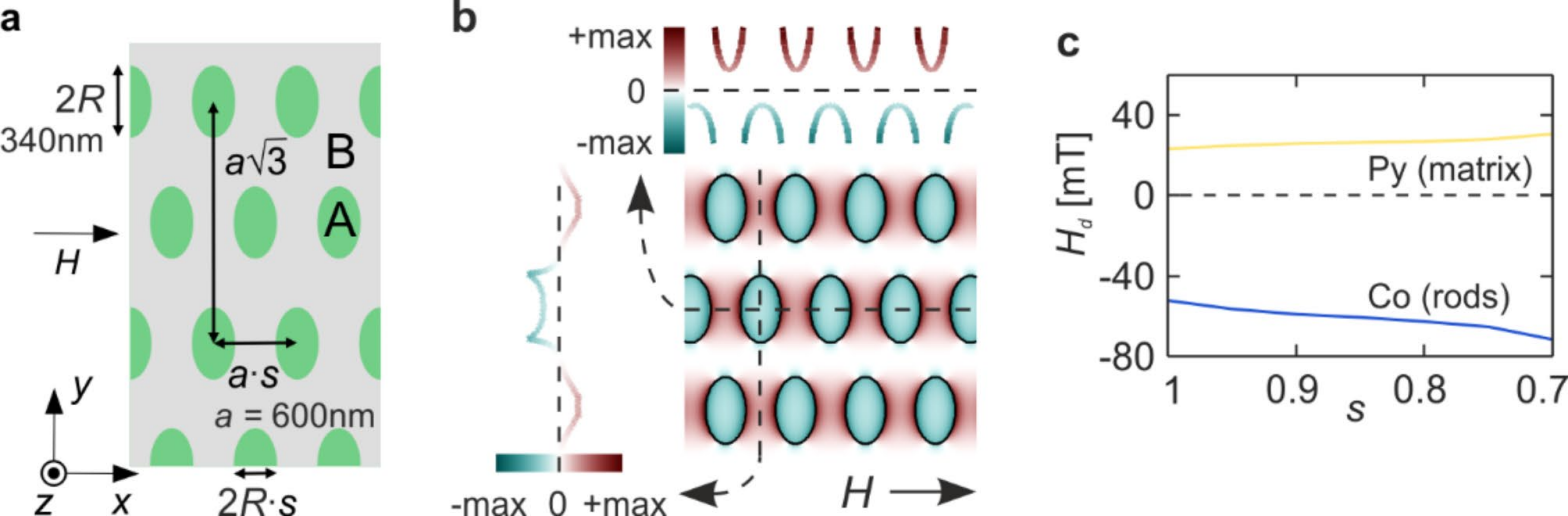
(**a**) The sketch of a 2D MC based on the squeezed hexagonal lattice: a thin-film matrix made from material B (Py) with dots of material A (Co) embedded in. The whole structure is squeezed in the *x*-direction (the direction of the external field *H*) by the structure ratio *s*. (**b**) Demagnetizing field for the squeezed Co/Py MC along with its cross-sectional profiles parallel (top) and perpendicular (left) to the external field. (**c**) The demagnetizing field in Py and Co plotted versus .

In this work, we consider a 2D MC in the form of a thin permalloy (Py) film, also known as the matrix, with periodically placed cobalt (Co) dots, as schematically shown in Fig. 1a. The thickness of the thin film is fixed at 30 nm and the material parameters used for calculations are: saturation magnetization 1.390e6 [A/m] in Co and 0.810e6 [A/m] in Py, exchange stiffness constant 2.8e-11 [J/m] in Co and 1.1e-11[J/m] in Py. The values of material parameters available in the literature are characterized by a certain scatter, so in our work we use exemplary values that fall within the range of those published in experimental studies^72–78^. Our initial structure is a hexagonal lattice with a lattice constant *a* = 600 nm. Circular dots with a diameter of 340 nm are placed at the nodes of this lattice. An external magnetic field *H* is applied in the plane of the MC along the *x*-direction. We consider a range of values of the external field sufficient to achieve magnetization saturation. What convinces us of the validity of such an assumption is the fact that all frequencies in the spectrum are significantly above zero, meaning that there are no Goldstone modes associated with the sample’s tendency to remagnetize.

In our studies, we employ the Plane Wave Method (PWM), which is a popular theoretical approach described in numerous works (see, e.g^50,78–81^). It allows for the calculation of spin wave frequencies and their profiles, i.e. spatial distributions of dynamic magnetization amplitudes. We use a version of this method based on the Landau-Lifshitz equation neglecting damping, as the damping little influence on the spin wave spectrum, as shown in Ref^28^.

In the structure under consideration, due to the shape of magnetic inclusions and the step change in magnetization between Co and Py, nonuniform demagnetizing field occurs at the dots-matrix interface^82,83^. In a material with higher saturation magnetization, i.e. Co, this field has a negative value, leading to a decreased effective magnetic field in the dots. On the other hand, in the Py matrix, which has lower saturation magnetization, the demagnetizing field has a positive value, resulting in an increased effective field. The distribution of the demagnetizing field can be calculated with use of the PWM (the detailed discussion of this topic is given in Refs^79,83^). For the squeezed Co/Py MC such distribution is shown in Fig. 1b. As can be seen, this field has its maximum absolute value at the material interface, but completely fills the interior of both dots and matrix areas between neighbouring dots (in the direction of the external field). Therefore, one way to enhance the demagnetizing field inside these areas may be to compress the entire structure along the direction of the external field – without changing the step in saturation magnetization at the material interface. Therefore, we introduce a structure ratio *s*, by which we multiply the *x*-coordinate of the compressed structure. Then, for *s* < 1, both the sizes of the dots and the distances between them are shortened in this direction (see Fig. 1a). The crystal sizes in the *y*-direction remain unchanged. It is worth noting that the filling fraction, i.e. the volume of cobalt in relation to the total sample volume, also remains unchanged. Naturally, *s* = 1 denotes the initial hexagonal structure. It should be noted that the compression of the structure leads to a change in the curvature of the boundary between the dot and the matrix, which also affects the value of the demagnetizing field. The resultant change in the value of the demagnetizing field accompanying the compression of the system is shown in Fig. 1c.

## Results and discussion

### Interaction of modes

**Fig. 2:**
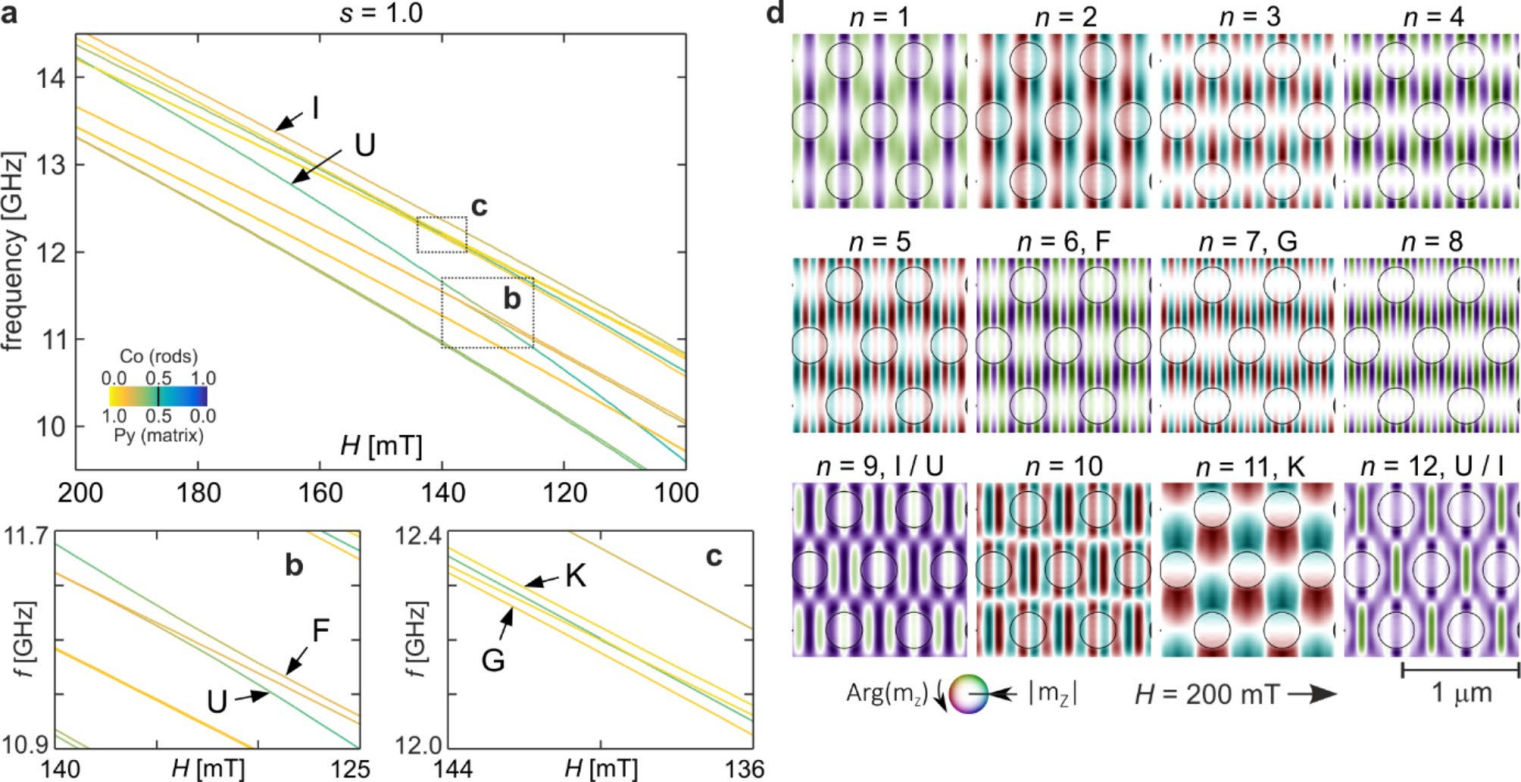
The spin-wave spectrum (twelve lowest-frequency modes) for cobalt dots in the permalloy matrix. (**a**) Frequency vs. external magnetic field (*H*) dependence. Line colours depict the in-Co/Py concentration according to the colour scale shown in the inset. (**b, c**) Enlarged regions indicated in (**a**) as rectangles b and c, respectively. (d) Spin-wave profiles of 12 lowest-frequency modes numbered sequentially in increasing order of frequency (*n* = 1, 2, 3, …) for *H* = 200 mT. Colours represent the phase, and intensity indicates the magnitude of dynamic magnetization, as shown in the inset at the bottom. Circles indicate the borders of the Co dots.

Figure 2 presents the results of calculations obtained for spin waves with zero wave vector (centre of the Brillouin zone) in the initial hexagonal structure, i.e. the structure without any squeezing (*s* = 1.0). It shows the frequencies and profiles for twelve modes with the lowest frequencies. Figure 2a illustrates the dependence of spin wave frequencies on the external magnetic field, obtained by decreasing its value in the range from 200 to 100 mT. The colours of the lines reflect the excitation energy concentration in one of the materials: yellow in Py, and blue in Co, according to the colour scale shown in the inset. The concentration is calculated with the use of the concentration coefficient introduced in Ref^45^. Of course, decreasing the external field results in lower spin wave frequencies, but it is characterized by an interesting trend. The frequencies of modes with strong concentration in Co dots decrease faster than those concentrated more in Py. This is especially clear for the two lowest (almost degenerate) states and the state labelled as U. This leads to a change in the order of modes in the spectrum, accompanied by the crossing of lines representing the frequencies of individual modes (hence their degeneracy) or their repulsion (anti-crossing), characteristic of hybridization phenomena. Due to the fact that the repulsion of states in the considered spectrum is small compared to the rate of frequency changes, and therefore not very visible, Fig. 2b and c show enlargements of two areas where mode repulsion occurs. The crossing of states in the spectrum indicates that spin waves do not interact with each other, whereas in the case of repulsion, we are dealing with interacting modes. It is this interaction between states with similar frequencies that leads to their hybridization, and therefore to the mixing of their profiles.

The profiles of the considered spin waves for an external field of 200 mT are shown in Fig. 2d. They are numbered according to the order in the frequency spectrum, i.e., *n* = 1 denotes the state with the lowest frequency, etc. Additionally, those modes that undergo hybridization in the considered range of the external field are labelled with letters of the alphabet according to their order for 200 mT (F for *n* = 6, G for *n *= 7, etc.), except for the mode labelled as U. This is a special mode, called the fundamental mode, which is the equivalent of the homogeneous state^84,85^. In the drawings depicting the profiles of spin waves, both the amplitude and the phase of the dynamic magnetization component’s precession are included. The phase is represented by colour, according to the colour scheme provided in the inset on Fig. 2d. The amplitude is indicated by the variation in intensity of that colour (white denotes the absence of precession). The application of an external magnetic field along the *x*-direction breaks the sixfold symmetry of the hexagonal lattice, so the system in the external field has a twofold symmetry. All profiles have the same symmetry (eventually with phase inversion).

**Fig. 3:**
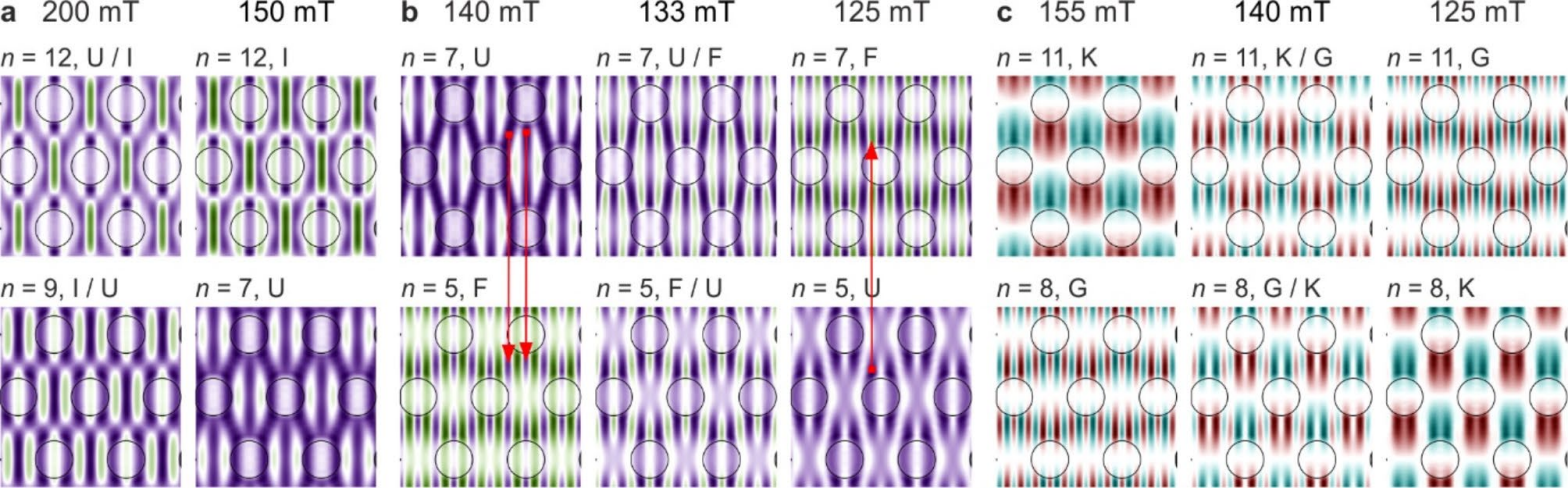
Evolution of spin-wave profiles of hybridizing modes. Please refer to the text for detailed description. The meaning of colours and symbols is the same as in Fig. 2d. The red arrows indicate the corresponding amplitude maxima.

As it is known, the fundamental mode is characterized by in-phase magnetization oscillations throughout the sample volume, which is not present in any of the profiles shown in Fig. 2d. This is because, at *H* = 200 mT, the fundamental mode (*n* = 12 hybridizes with mode *n* = 9, as indicated by labelling their profiles as I/U (U/I). As a result, these profiles are mixed. Of course, it is difficult to speak of a fundamental mode in this situation, as each of these profiles has characteristics of both coupled states. However, as the external field decreases, the hybridization weakens, and ultimately these states regain their separate characteristics. This is shown in Fig. 3a, where, in addition to the repeated profiles of these two states for *H* = 200 mT, their profiles for *H* = 150 mT are also plotted. Due to the interaction and associated hybridization, modes switch places in the spin wave spectrum - even though their frequencies do not intersect.

An attentive observer may notice that the profile of state U for 150 mT, shown in Fig. 3a, contains small areas of opposite phase. This is the result of another hybridization that the fundamental mode undergoes, reaching its maximum at *H* = 133 mT. This is a part of the spectrum marked in Fig. 2a by the rectangle labelled as b and shown in detail in Fig. 2b. This time, mode U hybridizes with mode F, which in the field range between 140 and 133 mT, after crossing with a lower state, is the fifth in the spectrum (*n* = 5). The evolution of the profiles of interacting states is presented in Fig. 3b. For the state *n* = 7 (U), small areas of opposite phase visible for 140 mT are stretched in the *y*-direction in the entire matrix (*H* = 133 mT), to then also appear inside the dots for *H* = 125 mT, reproducing the character of state F (*n* = 5 for *H* = 140 mT). At the same time, for the state *n* = 5, these areas gradually disappear. For 133 mT, their intensity is already very low, indicating a small amplitude of precession in these areas. With further decreasing of the external field, precession in these areas is completely extinguished, and mode *n* = 5 acquires the characteristic of the fundamental mode (precession of magnetization in the same phase over the entire area). Thus, as a result of interaction and the related hybridization, modes swap places in the spin wave spectrum - even though their frequencies do not intersect. This swapping also results in a change in cobalt concentration, which is visible in the frequency spectrum (colour of lines F and U in Fig. 2b).

In the considered spectrum, there is another case of hybridization – between modes G and K. The interaction of these states occurs in a fairly wide range of fields around 140 mT (hybridization maximum). The centre of this range is marked in Fig. 2a by the rectangle labelled as c and shown in detail in Fig. 2c. The repulsion of these states in the spectrum is very weak, and therefore almost imperceptible. There are also no noticeable changes in concentration coefficient (line colour in the spectrum). However, the analysis of profiles (Fig. 3c) shows a clear, gradual mixing of them with a maximum at *H* = 140 mT, and then a swapping of places – state *n* = 8 in a higher field has a profile G, and in a lower one, K, while state *n* = 11 is the opposite, despite the lack of intersection of these states in the spectrum. This blending of profiles is much earlier visible for mode K, as it contains larger areas of the same phase compared to mode G (it is also worth comparing its profiles from Fig. 3c with the profile for 200 mT, Fig. 2d).

The change in the order of modes in the spin wave spectrum with a change in the external magnetic field can also occur due to their crossing, instead of hybridization. Whether modes whose frequencies are approaching each other will cross or hybridize depends on whether they interact with each other or not. This, in turn, is closely related to matching their profiles. Let’s go back to Fig. 2d, where modes K and G are far from hybridization. The profile of mode K in the direction perpendicular to the external field (*y*, down-up direction in the figure) has nodal lines passing through the centres of the Co dots and distinct amplitude maxima with alternating phase in the areas between neighbouring rows of dots. In this direction (*y*), mode G (*n* = 7) also possesses a similar property, as well as mode with the number *n* = 8 (lower modes, although some of their profiles also exhibit this feature, have substantially lower frequencies throughout the examined range of external fields and can therefore be excluded from these considerations). In the direction parallel to the external field (*x*, left-right direction in the figure), mode K has one nodal line between neighbouring rows of dots and anti-nodal lines (amplitude maxima) in the middle of these rows, with these maxima having opposite phases. Therefore, we have one half-wave between neighbouring rows of dots in this direction. To some extent, the situation is similar for mode G. Analysing its profile in the direction *x*, we also have amplitude maxima at the centres of neighbouring rows of dots, and this time they have opposite phases as well. However, in this case, there are five half-waves between them (five nodal lines and four consecutive maxima with alternating phases). Thus, the occurrence of matching amplitude maxima for modes K and G in the same MC areas causes their coupling when their frequencies approach each other. (Not all maxima have to match each other. Some are sufficient, but the phase consistency is important.) The situation is different for mode number 8. In its profile, there are also 5 half-waves in the direction *x*between neighbouring rows of dots, similar to mode G, but this time we have nodal lines at the centres of these rows, not amplitude maxima. It is worth noting that similar properties have been found for the spectrum of normal modes of 2D magnetic dots and rings in a vortex state^18,19,86,87^(see especially Fig. 8 in Ref^86^).

Let’s focus on the phase of the hybridizing modes. Available results in the literature for simple systems 1D^88^and quasi-1D^19,86,87^ may suggest that an anti-phase matching is preferred. In 2D systems, the patterns of most profiles are rather complex, making it often difficult to say with certainty whether hybridization occurs for maximum amplitude matching in phase or in anti-phase. Consider, for example, the relatively simple case of the profiles in Fig. 3b, where the corresponding amplitude maxima are indicated by red arrows. For an external field of 140 mT, the maxima of profile U correspond to the maxima of profile F in the same phase. Consequently, reducing the external field leads to hybridization. However, for a field of 125 mT, the corresponding maxima have opposite phases. Of course, increasing the external field from this value also leads to the same hybridization. Thus, the obtained results show that matching maxima in phase or anti-phase does not matter. In the considered case, the phase of mode F, after passing through the range of fields in which it couples with mode U, is restored (see profile F for 140 and 125 mT in Fig. 3b). The phase of mode U also remains unchanged, but the amplitude maxima are shifted to regions where previously the magnetization precession was suppressed (see profile U for 140 and 125 mT in Fig. 3b). The situation is different in Fig. 3c. Here, one of the modes (K) retains its phase during the transition through hybridization, while the phase of the second mode (G) is reversed. The transition through hybridization does not change the character of the modes, i.e., their quantization, in all cases shown here. It can also be observed that, for the maximum mixing of the hybridizing modes profiles, they largely have opposite phases.

In experimental methods such as time-resolved scanning transmission x-ray microscopy (TR-STXM)^89^and time-resolved magneto-optical Kerr effect (TR-MOKE)^90^, the spatial distribution of dynamic magnetization is observed, specifically the spin wave profile – both amplitude and phase. Thus, these methods allow for the direct observation of the mixing of mode profiles associated with their hybridization. Moreover, in ferromagnetic resonance (FMR), the hybridization of certain modes should also be observable. In FMR, the intensity of the resonance line is proportional to the square of the average amplitude of the spin wave, meaning that modes with antisymmetric profiles are not observed^91^. Therefore, the best candidate for observation via FMR is the quasi-uniform mode, referred to as U. The hybridization of this mode and the associated evolution of the profile with changes in the external field is particularly interesting. As the external field changes, the profiles mix (Fig. 3b), which in turn alters the size of areas with opposite phases – for both hybridizing modes. These changes appear smoothly and result in the intensity of the strong line in the FMR spectrum progressively decreasing while the intensity of the weak line increases. For a certain field (133 mT for Fig. 3b), these intensities will equalize and then swap places. Such behaviour of the FMR signal does not necessarily indicate the hybridization of the quasi-uniform mode, but if such hybridization occurs, it should affect the FMR spectrum in this way.

### Demagnetizing field enhancement

**Fig. 4:**
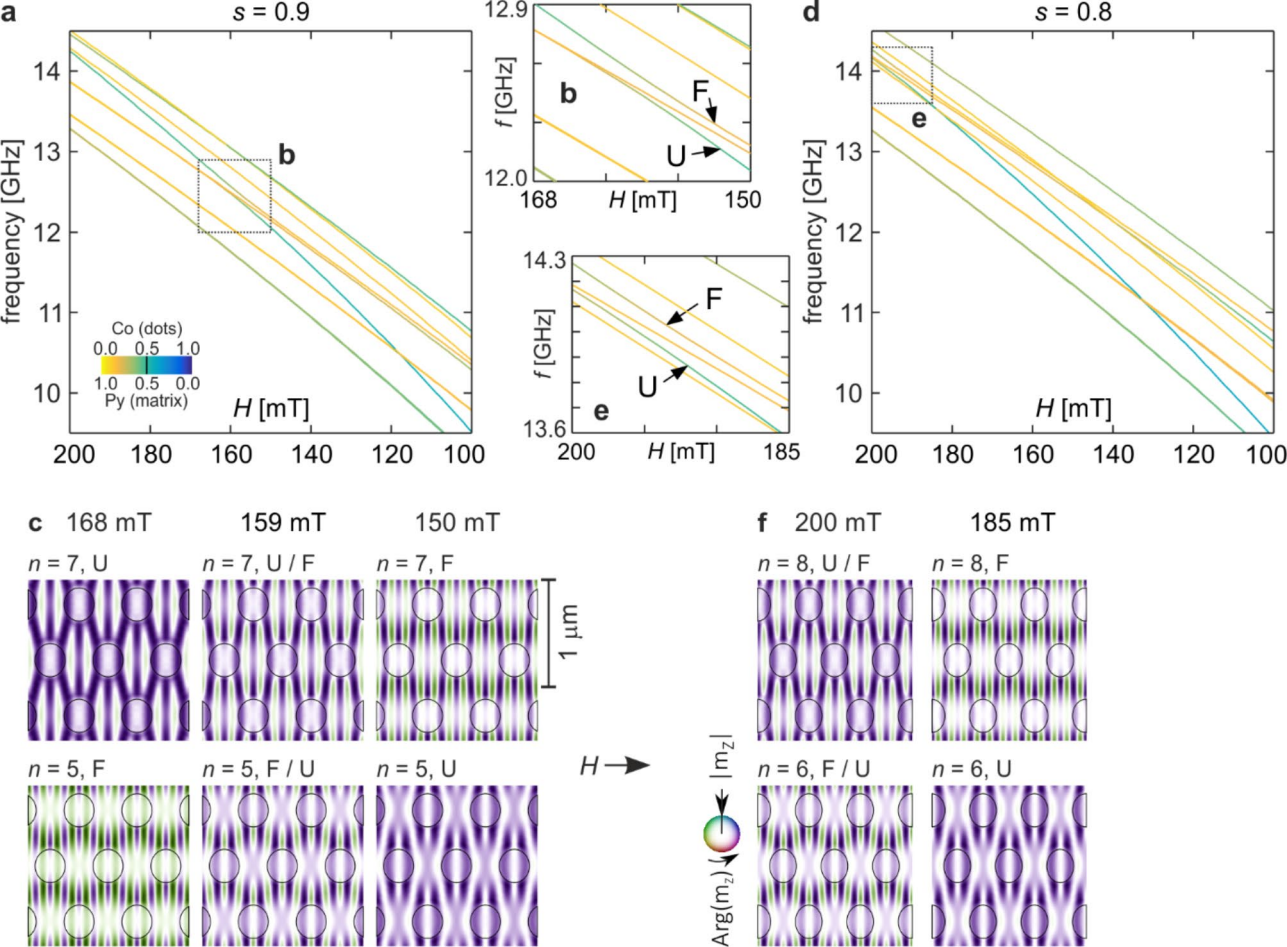
Ten lowest frequencies of spin waves for two squeezed Co/Py MCs: (a-c) for *s* = 0.9 and (d-f) for *s* = 0.8. (a, d) Frequency vs. external magnetic field (*H*) dependencies. Line colours depict the in-Co/Py concentration according to the colour scale shown in the inset in (a). (b, e) Enlarged regions of the spectra indicated in (a) as a rectangle b and in (d) as a rectangle e, respectively. (c, f) Evolution of spin-wave profiles of hybridizing modes (see the text for detailed description). The colours represent the phase, and the intensity indicates the magnitude of the dynamic magnetization, as shown in the inset in (f). The ellipses indicate the borders of the Co dots.

Squeezing of the structure (dimension reduction) parallel to the direction of the external field leads to strengthening of the demagnetizing field, as we show in Fig. 1c and discuss in Section “The model and the method”. Figure 4a shows the dependence of frequency on the external field change for a squeezed structure with a ratio *s* of 0.9. The frequency scale is the same as in Fig. 2a for the base structure (*s* = 1.0). The general tendency is similar to the uncompressed structure, i.e. the frequencies of modes strongly localized in Co (blue colour) decrease faster than those localized in Py (yellow colour). One of the main differences is that for the compressed structure, the modes mainly concentrated in the Py matrix have shifted up in the frequency spectrum, while the modes concentrated more in the Co dots have hardly changed their positions. This phenomenon is a result of the interplay of two effects. One is the reduction of linear dimensions in the *x*-direction. This leads to an increase in the frequency of modes quantized in this direction (see Fig. 2d), similar to what happens in a potential well. The second effect is the strengthening of the demagnetizing field. In permalloy, this causes an increase in the effective magnetic field, and consequently, an additional increase in the frequencies of modes mainly excited in it. In cobalt, its influence is opposite - it causes a reduction in the effective field, thus counteracting the frequency increase resulting from the reduction in dimensions for modes with high concentration in Co.

The mutual shift of the spectra of these two groups of states results in the shift of the hybridization of individual pairs of modes towards higher fields. In the considered range of values of the external field, there is only one case of mode coupling. These are, as in the case of *s* = 1.0, modes U and F, whose maximum hybridization occurs at around 159 mT, i.e. shifted by about 36 mT compared to the uncompressed structure. The spectrum range in which this hybridization occurs is marked with rectangle b in Fig. 4a, and is plotted in detail in Fig. 4b. The gradual mixing of the U and F mode profiles, resulting in a change in their order in the spectrum, is similar to that of the original structure (Fig. 4c). However, there is no hybridization of U and I modes here, because already for *H* = 200 mT, mode U is below mode I in the spectrum to the extent that they do not interact. The same goes for modes G and K. Their order in the spectrum is already reversed at 200 mT. It is worth noting that squeezing the system along the *x* direction does not introduce an additional breaking of symmetry because it is applied along the same direction as the external field. Thus, in compressed structures, the profiles maintain a twofold symmetry.

Further compression of the structure (*s* = 0.8) does not bring qualitative changes, but it causes further shifting of the states in the spectrum, and consequently, also a shift of hybridization towards higher external fields. Figure 4d shows the dependence of the spectrum on the external field for a structure ratio of 0.8. The region where hybridization of U and F modes occurs is marked with rectangle e and shown in detail in Fig. 4e. Mixing of their profiles and the subsequent change in the order of modes in the spectrum is shown in Fig. 4f. The maximum of this hybridization occurs at an external field of about 200 mT. It is a value close to the hybridization observed for the base structure, only this time a different pair of states hybridizes in a similar range of fields (with one of them being the same fundamental mode).

**Fig. 5:**
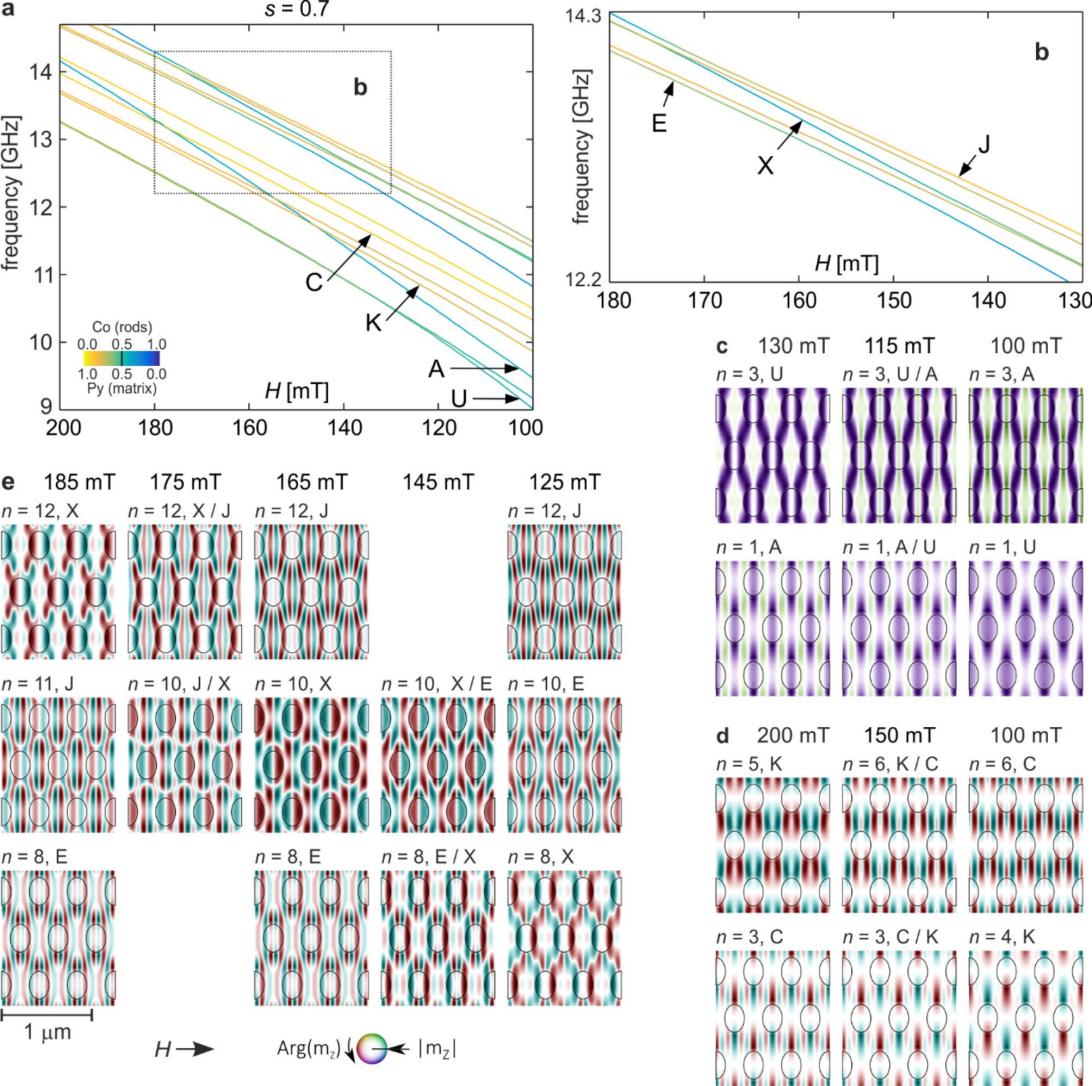
Twelve lowest frequencies of spin waves for the squeezed Co/Py MC (*s* = 0.7). (a) Frequency vs. external magnetic field (H) dependence. Line colours depict the in-Co/Py concentration according to the colour scale shown in the inset. (b) Enlarged region of the spectrum indicated in (a) as a rectangle b. (c-e) Evolution of spin-wave profiles of hybridizing modes (see the text for detailed description). The colours represent the phase, and the intensity indicates the magnitude of the dynamic magnetization, as shown in the colour scale at the bottom. The ellipses indicate the borders of the Co dots.

The results obtained for the structure ratio *s* = 0.7 are shown in Fig. 5. The frequency spectrum as a function of external field shows several cases of mode interactions (Fig. 5a). Primarily, the fundamental mode undergoes hybridization once again. This time, for fields in the range of 120 − 100 mT, it interacts with mode A, which was the lowest frequency state for the base structure and the field of 200 mT (cf. Figure 2d). As a result of the typical mixing of profiles and the subsequent state swapping, for *H* < 100 mT, the fundamental mode obtains the lowest frequency (see Fig. 5c). Furthermore, due to repulsion from mode A, its frequency rapidly decreases with decreasing external field, much faster than the other states. Therefore, it is a good candidate for being the Goldstone mode, which in low fields achieves a zero frequency and is responsible for magnetization reconfiguration^92^, similar to what happens in 1D MCs^25^. As shown in the case of 2D magnetic dots^93–95^, the profile of this mode indicates the areas of the sample that are particularly susceptible to magnetization reversal, from which this process begins. These are the areas of the highest dynamic magnetization of the Goldstone mode. Considering the increasing concentration in cobalt for mode U, it can be inferred that the magnetization reversal process in the studied magnonic systems should start from cobalt dots.

Another pair of interacting states are modes K and C. The evolution of their profiles resulting from hybridization is plotted in Fig. 5d. As can be seen, they are mixed throughout the entire range of external fields, especially noticeable in the upper profiles. Interestingly, in the spectrum from Fig. 5a, the frequency dependence of these modes on the external field appears to be parallel, making it difficult to observe the typical repulsion between them that occurs during hybridization. This is an expression of a general tendency that also occurs for the other examples analysed in this study. The wider the range of external field values for which modes are coupled, the weaker, and therefore less visible, the repulsion of their frequencies in the spectrum.

Let us return to our considerations, regarding the matching of profiles as a condition for mode coupling. In the *y* direction, mode C has distinct amplitude maxima with alternating phases in the areas between neighbouring rows of dots and nodal lines passing through the centres of these rows, similar to mode K (compare their profiles in Fig. 2d). In the *y* direction for mode C, we have three half-waves with maxima of opposite phases at the centres of adjacent rows of Co dots. Thus, similarly to mode G previously, also for mode C, (some) maxima of its amplitude are located in the same areas of the sample as the maxima of mode K.

In Fig. 5e, the evolution of the profile of mode X is presented. We have adopted the convention according to which the alphabetical designations of modes correspond to their order in the spin wave spectrum for the initial parameters, i.e., for the base structure and the external field of 200 mT. Mode X is then relatively high in the spectrum, above the twentieth mode, hence its designation. Due to the compression of the MC structure, similar to the other modes with high cobalt concentration, it descends further compared to the other states. Ultimately, for *s* = 0.7, it is positioned low enough (*n* = 12 for 185 mT) to interact with mode J in the field range between 185 and 165 mT. Of course, as in other cases, these modes swap places in the frequency spectrum. Thus, further decreasing the value of the magnetic field leads to the interaction of mode X with the next lower mode with similar symmetry (E). As a result of this subsequent hybridization for a field of 125 mT, mode X takes the place of mode E in the spectrum (*n* = 8).

It is interesting that the profile of mode X between the maxima of these two hybridizations (*n* = 10 for 165 mT, i.e., the middle row and middle column in Fig. 5e) is completely different than before the first hybridization and after the second one (profiles *n* = 12 for 185 mT and *n* = 8 for 125 mT are almost identical - except for a uniform phase reversal of π). This may be the result of simultaneous, albeit weak, interaction with both modes J and E. Such interpretation may be supported by the fact that, although modes J and E do not directly couple with each other, eventually there is a transfer of certain characteristics between them. For a field of 185 mT, the profile of mode J (*n* = 11) has significantly higher amplitude of Co dots than the profile of mode E (*n* = 8). However, for a field of 125 mT, the situation is reversed - the profile of mode E (*n* = 10) shows stronger magnetization oscillations in cobalt than the profile of mode J (*n*= 12). Such three-mode hybridization was observed in vortex state nano-dots, where its effects were similar to those described above, i.e., an exchange of certain characteristics between the profiles of two modes that do not directly interact, but through a third mode^19^.

## Conclusion

In the studied two-dimensional magnonic crystals of cobalt/permalloy, a change in the value of the external magnetic field causes a non-uniform shift in the frequency of spin waves. This shift, roughly speaking, depends on the concentration of a given mode in cobalt. As a result, modes with similar symmetries approach each other (or move apart), leading to their coupling and the accompanying hybridization (or its disappearance due to decoupling).

Squeezing of the 2D MC structure along the direction of the external magnetic field enhances the demagnetizing field, resulting in a change in the effective field. This effect is opposite in Co dots compared to Py matrix. The change in the effective field causes additional shifts in the frequency of spin waves - also non-uniform, depending on which material the individual modes are more concentrated in. Consequently, we obtain a shift in the hybridization of a specific pair of modes towards higher values of the external field. This provides a potential opportunity to design (within a certain limited range) which pairs of modes will be coupled in a specific range of external fields, or for what range of field values a coupling of a specific pair of modes will occur.

In one of the considered cases, we observed the simultaneous coupling of two different states, which do not interact directly with each other, with a third state. Although this coupling is very weak, it leaves a trace in the profiles of these states, which do not interact directly with each other, but only through the third state. A similar three-mode hybridization effect occurs for normal modes in finite 2D dots with vortex magnetization. Another effect observed in our work, which also occurs in 2D vortex states, is the multiple hybridization of the fundamental mode. In the case of 2D dots, both of these effects occur as a result of the competition between exchange and dipolar interactions. In the ferromagnetic magnonic crystals studied by us, these effects are a consequence of manipulating the demagnetization field, the existence of which is a result of dipolar interactions. Thus, we can venture to make a general conclusion that they should also occur in other systems where similar competing interactions are present.

## Data Availability

Data sets generated during the current study are available from the corresponding author on reasonable request.
